# 750 V Breakdown in GaN Buffer on 200 mm SOI Substrates Using Reverse-Stepped Superlattice Layers

**DOI:** 10.3390/mi15121460

**Published:** 2024-11-30

**Authors:** Shuzhen You, Yilong Lei, Liang Wang, Xing Chen, Ting Zhou, Yi Wang, Junbo Wang, Tong Liu, Xiangdong Li, Shenglei Zhao, Jincheng Zhang, Yue Hao

**Affiliations:** 1 Guangzhou Wide Bandgap Semiconductor Innovation Center, Guangzhou Institute of Technology, Xidian University, Guangzhou 510555, China; yllei0418@163.com (Y.L.); wangliang20210618@163.com (L.W.); 13571991665@163.com (T.Z.); wy13412581799@163.com (Y.W.); wjb874085975@163.com (J.W.); tongliu0616@163.com (T.L.); lixiangdong28@126.com (X.L.); slzhao@xidian.edu.cn (S.Z.); jchzhang@xidian.edu.cn (J.Z.); yhao@xidian.edu.cn (Y.H.); 2 State Key Laboratory of Wide Bandgap Semiconductor Devices and Integrated Technology, School of Microelectronics, Xidian University, Xi’an 710071, China; 3 Advanced Microelectronics Institute, Xidian-Wuhu Research Institute, Wuhu 241008, China; chenxing@xdwh-inst.com

**Keywords:** GaN-on-SOI, reversed stepped superlattice buffer, metal–organic chemical vapor deposition

## Abstract

In this work, we demonstrated the epitaxial growth of a gallium nitride (GaN) buffer structure on 200 mm SOI (silicon-on-insulator) substrates. This epitaxial layer is grown using a reversed stepped superlattice buffer (RSSL), which is composed of two superlattice (SL) layers with different Al component ratios stacked in reverse order. The upper layer, with a higher Al component ratio, introduces tensile stress instead of accumulative compressive stress and reduces the in situ curvature of the wafer, thereby achieving a well-controlled wafer bow ≤ ±50 µm for a 3.3 µm thick buffer. Thanks to the compliant SOI substrate, good crystal quality of the grown GaN layers was obtained, and a breakdown voltage of 750 V for a 3.3 µm thick GaN buffer was achieved. The breakdown field strength of the epitaxial GaN buffer layer on the SOI substrate is estimated to be ~2.27 MV/cm, which is higher than the breakdown field strength of the GaN-on-Si epitaxial buffer layer. This RSSL buffer also demonstrated a low buffer dispersion of less than 10%, which is good enough for the further processing of device and circuit fabrication. A D-mode GaN HEMT was fabricated on this RSSL buffer, which showed a good on/off ratio of ~10^9^ and a breakdown voltage of 450 V.

## 1. Introduction

Gallium nitride (GaN)-based power devices have the advantages of a large bandgap, high electron mobility, and high breakdown voltage, which makes them highly desirable for high-efficiency power conversion systems [1,2,3]. The quality of GaN materials is critical for achieving superior device performance. Native GaN bulk substrates are considered ideal for GaN epitaxy because of their high quality, characterized by low dislocation density and low impurity concentrations [4]. However, GaN bulk substrates are very expensive and are available only in diameters smaller than 100 mm. This limitation necessitates the use of heteroepitaxy of GaN on foreign substrates. Considering economic factors, the low-cost Si substrates are more attractive compared to SiC substrates [5]. In order to unlock the full potential of GaN high-electron mobility transistors (HEMTs) for power applications, monolithic integrated GaN ICs are essential to enable faster switching than power systems using discrete components by suppressing parasitic inductance and capacitance.

However, the monolithic integration of power devices on GaN-on-Si substrates suffers from the crosstalk between devices and the “backgating” effect through “substrate contact”. One of the solutions to suppress the crosstalk and “backgating” effect is the use of an SOI (silicon-on-insulator) substrate combined with trench isolation [6,7]. GaN-on-Si devices exhibit a “backgating” effect, where the silicon substrate influences the channel’s electrical properties in GaN-on-Si structures. This effect can lead to instability in the threshold voltage and leakage current, affecting device performance and reliability [8]. An alternative solution is the use of engineered substrates with a poly-AlN core, commercially available as QST^®^ (QROMIS Substrate Technology) [9,10,11,12]. The relatively high price of QST^®^ substrate hinders its widespread application. Recently, great interest has been raised for GaN-on-Sapphire, which can also serve as substrates of GaN monolithic integration circuits. The drawback of GaN-on-Sapphire is the low thermal conductivity of sapphire substrates [13,14,15]. Therefore, the SOI substrate provides a good trade-off between the low cost and high performance of monolithic circuits.

The flexible SOI substrate structure is composed, from bottom to top, of a handle silicon wafer, a buried oxide (BOX) layer that serves as electrical insulation, and a top silicon semiconductor layer. GaN growth on compliant SOI substrates using metalorganic chemical vapor deposition (MOCVD) was first reported in [16] for a higher crystal quality than growth directly on Si substrates. This quality improvement of the epitaxial layer was attributed to the compliant effect that the top silicon overlay and the buried SiO_2_ layer of the SOI substrate absorb most of the strain. Simoen et al. in [17] reported a defect assessment in AlN nucleation layers grown on silicon and SOI substrates and indicated a lower trap density for the layers on SOI, suggesting an AlN layer with better quality.

Although GaN materials grown on SOI can achieve better morphology, how to adjust the wafer warpage and stress control during epitaxy remains a key consideration that needs to be addressed. The design of buffer layers has long been a key focus in the research on GaN heteroepitaxy. Buffer layer strategies such as AlN buffer layers [18], AlGaN aluminum composition gradient buffer layers [19], and superlattice structures [20] have been adopted successively, all aimed at improving the quality of GaN epitaxial layers. The importance of the buffer layer design lies not only in optimizing lattice quality but also in its impact on vertical breakdown characteristics. For high-performance devices, vertical breakdown capability is crucial. Typically, there is a positive correlation between the thickness of the buffer layer and the breakdown voltage. Therefore, one of the current goals is to further increase the substrate size and the stacking thickness of GaN on SOI. However, the growth of thick GaN layers for high breakdown voltage devices on compliant SOI substrates remains a challenge in terms of warpage control and mechanical control for thick buffers [21]. Simply increasing the buffer thickness is not a feasible method, as the intrinsic curvature of the wafer may approach or even exceed the upper limit of the plastic deformation curvature as the buffer thickness increases. This is an undesirable situation, as it could lead to the cracking of the epitaxial wafer. To deal with the situation, Imec proposed the reversed stepped superlattice (RSSL) structure, which is composed of two superlattice (SL) layers with different Al component ratios stacked in a reverse order. The upper layer, with a higher Al component ratio than the lower layer, introduces tensile stress and reduces the in situ curvature of the wafer, thereby achieving the goal of reducing warpage [22,23]. Following this proposal, we grow the GaN buffer layer of the RSSL structure in this work using MOCVD growth and demonstrate a breakdown voltage of 750 V for a 3.3 µm thick GaN buffer.

## 2. Epitaxy and Device Fabrication

**Figure 1:**
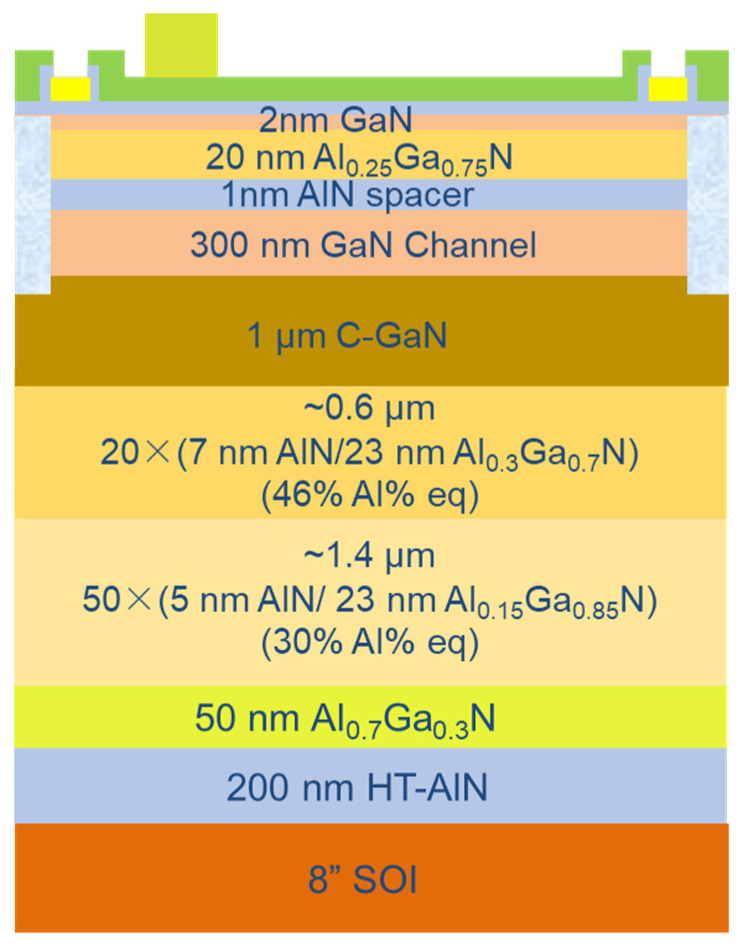
Schematic diagram of the GaN epitaxial wafer structure.

In this work, the SOI substrate’s top layer is P-type silicon (111) with a thickness of 2 µm and a resistivity of 0.01–0.025 Ω·cm; the BOX layer is SiO_2_ with a thickness of 1 µm; and the handle layer is silicon (100) with a thickness of 1070 µm. The GaN-on-SOI buffer structure was grown on 200 mm SOI substrates in the AMEC Prismo 480-PD chamber, using MOCVD with Ga, Al, and N sources of trimethylgallium (TMGa), trimethylaluminum (TMAl), and ammonia (NH3), respectively, using carrier gases of hydrogen (H2). The buffer scheme was designed, as shown in Figure 1, and has a 200 nm AIN nucleation layer, a 50 nm AlGaN lower transition layer, a first superlattice SL1 formed by repeating 5 nm AIN/23 nm Al_0.15_ Ga_0.85_N unit cells 50 times (equivalent average aluminum content: Al% = 30%), a second superlattice SL2 formed by repeating 7 nm AIN/23 nm Al_0.3_Ga_0.7_N unit cells 20 times (equivalent average aluminum content: Al% = 46%), a 1 µm carbon-doped GaN layer, a 300 nm GaN channel layer, a 1 nm AlN insertion layer, a 20 nm Al_0.25_Ga_0.75_N barrier layer, and a 2 nm GaN cap layer. The ohmic contacts were processed using a Ti/Al/Ni/Au (20/140/55/45 nm) stack and annealed at 830 °C in an N_2_ environment for 30 s. Then, multiple N ion implantation procedures were performed to achieve lateral isolation. Subsequently, 2.5 nm of Al_2_O_3_ and 50 nm of SiO_2_ were deposited sequentially by PEALD and PECVD, respectively. The gate metal of the device consists of 20 nm/120 nm Ni/Au. After depositing the gate metal, CF_4_, Ar, and N_2_ gases were used for reactive ion etching (RIE) to etch SiO_2_ and open the source/drain windows. The Al_2_O_3_ layer served as the etch stop layer for RIE, and after SiO_2_ was etched, it was removed by soaking in diluted HCl for 1 min. The fabricated HEMT has a gate width (Wg) of 100 µm, a gate length (Lg) of 1 µm, a gate-to-source distance (Lgs) of 0.75 µm, and a gate-to-drain distance (Lgd) of 6 µm.

## 3. Results and Discussion

**Figure 2:**
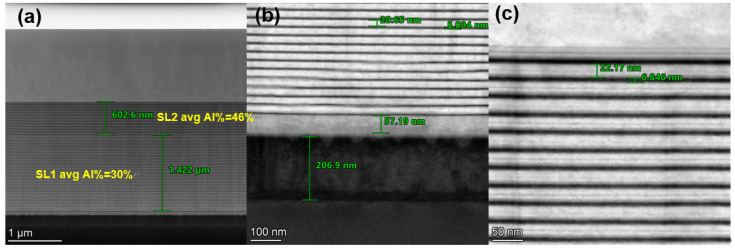
(**a**) TEM cross-sectional image of the epitaxial layer with a thickness of ~3.3 µm, using an RSSL buffer. (**b**) Local TEM magnification of SL1 and (**c**) local TEM magnification of SL2.

**Figure 3:**
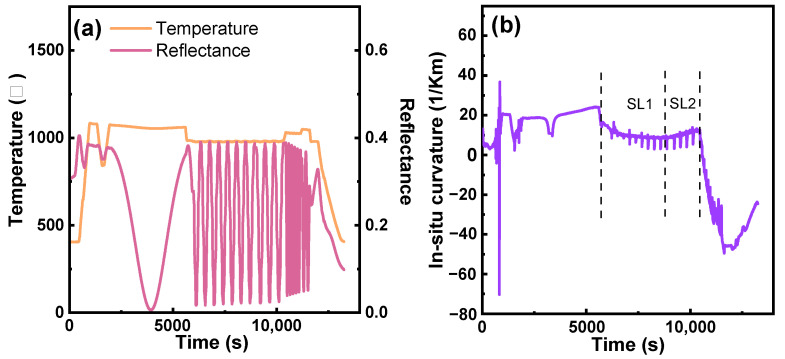
Growth curve of the buffer: (**a**) reflectance and growth temperature and (**b**) in situ wafer curvature.

Figure 2 shows a TEM cross-section image of the grown (Al) GaN stack, with a total thickness of ~3.3 µm, using an RSSL buffer. In this RSSL buffer, SL2 (of average Al percentage of 46%) was stacked on top of SL1 (of average Al percentage of 30%). Figure 3 shows the buffer growth curves of reflectance, growth temperature, and the in situ wafer curvature. It is seen in Figure 3b that the slope of the growth curvature was changed from negative in SL1 to positive in SL2, indicating that SL2 with higher Al content introduces tensile stress instead of accumulative compress stress in a conventional stepped superlattice stack. Table 1 shows the in situ warpage of the post-epi wafer on 200 mm SOI substrates, indicating that the in situ warpage of the wafer is well controlled within the ±50 µm range, fully meeting the specification of bow ≤ ±50 µm; the wafer warpage test demonstrates that the RSSL structure can effectively adjust the intrinsic curvature of the wafer. Following this RSSL approach, no wafer breakage has been observed during the epitaxial growth in our MOCVD chamber or our pilot line of device processing.

GaN layers with good crystal quality were obtained, as illustrated by the X-ray diffraction full-width half maximum (XRD FWHM) values in Table 2. Table 2 shows the XRD FWHM values of the GaN-on-SOI sample at four positions. This also reflects good consistency.

**Figure 4:**
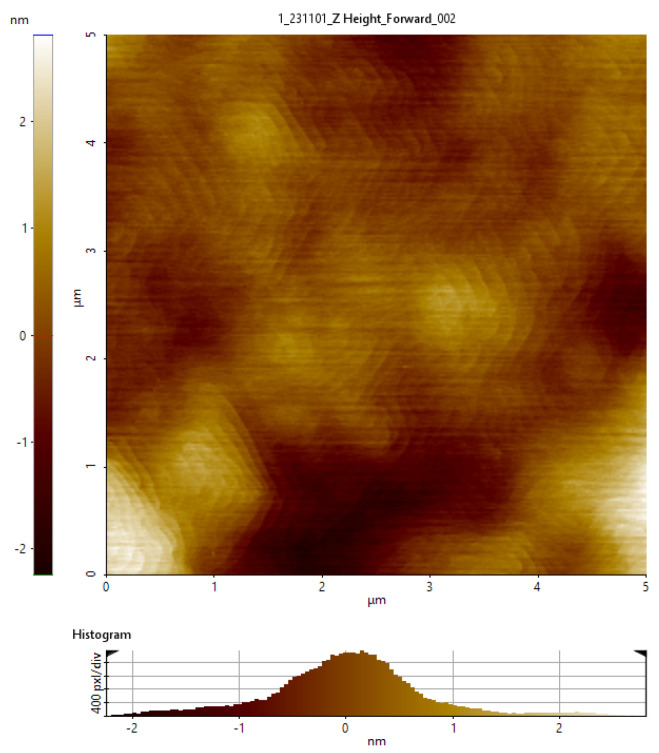
AFM test of surface roughness of 200 mm GaN-on-SOI.

**Figure 5:**
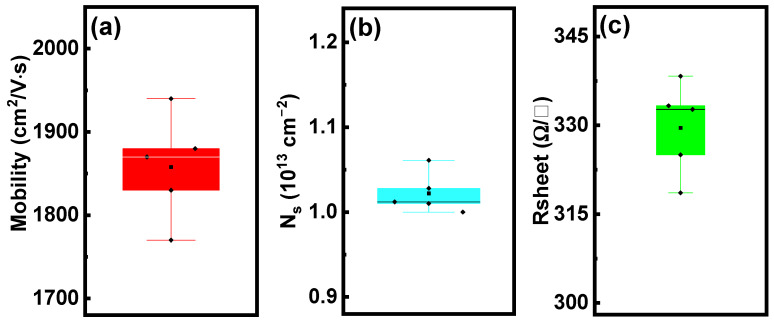
(**a**) 2DEG electron mobility, (**b**) 2DEG concentration, and (**c**) 2DEG sheet resistance.

Scanning atomic force microscopy (AFM) was used to measure the surface morphology of the GaN epitaxial layer with a scanning size of 5 µm × 5 µm, as shown in Figure 4 The AFM image shows a smooth and flat surface, with an RMS value of 0.7 nm. The smooth epitaxial layer surface is beneficial for the 2DEG performance and subsequent wafer processing. Figure 5 shows that the AlGaN/GaN heterojunction based on this stack has excellent electrical properties, with a 2DEG mobility and concentration of 1850 cm^2^/V·s and 1.03 × 10^13^ cm^−2^, respectively, at 300 K, and a 2DEG sheet resistance of 330 Ω/□.

**Figure 6:**
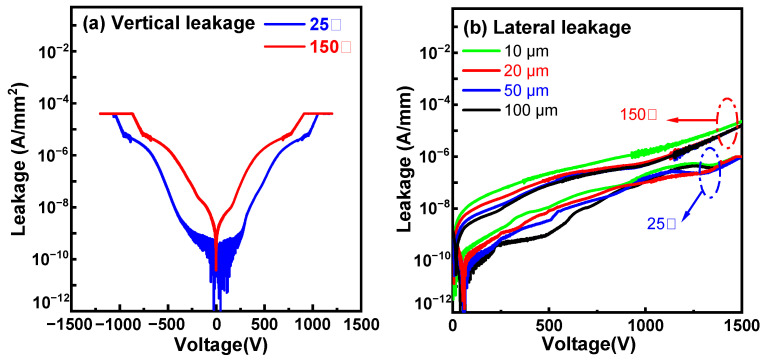
(**a**) vertical and (**b**) lateral leakage curves of the buffer on 200 mm GaN-on-SOI substrates.

**Figure 7:**
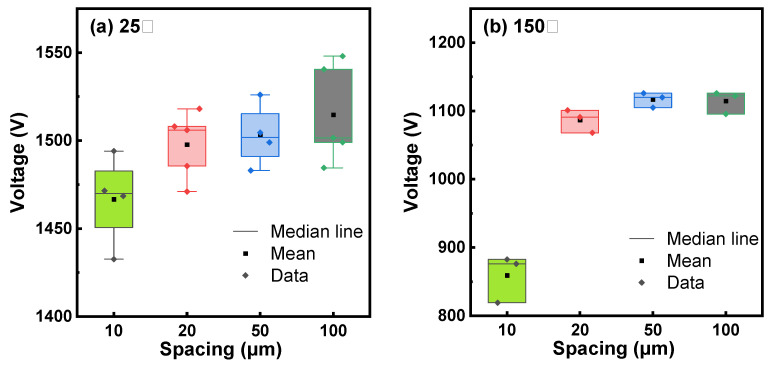
Voltage values for different isolation spacings on GaN-on-SOI with RSSL buffer layers when the current reaches 1 × 10^−6^ A/mm: (**a**) tested at a temperature of 25 °C and (**b**) tested at a temperature of 150 °C.

**Figure 8:**
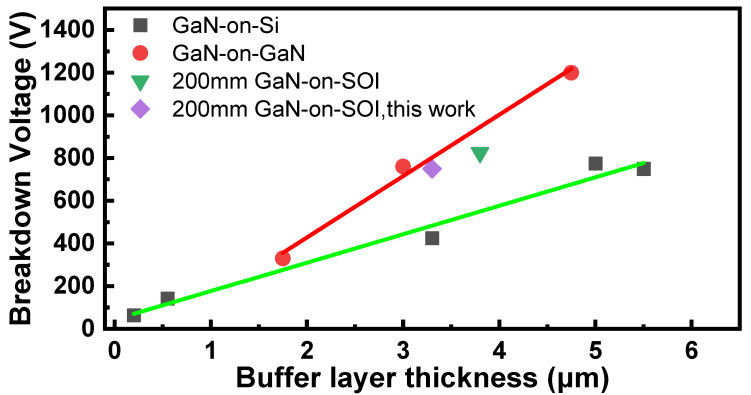
Breakdown voltage of GaN epitaxial layers with different buffer layer thicknesses on different substrates [6,24,25,26] (including Si [24], GaN [26], SOI [6]).

Vertical buffer leakage was measured by grounding the substrate and biasing the top ohmic anode with a positive or negative voltage. Lateral buffer leakage was measured by applying a bias between two ohmic contacts with an isolation gap between them. From the leakage curves in Figure 6a, a vertical breakdown voltage (BV) of ~900 V (@10 × 10^−6^ A/mm^2^) was extracted at 25 °C. It is known that the box in the SOI substrate can withstand 100 V; thus, the sole epitaxial buffer layer withstands ~800 V. The lateral buffer leakage curves at 25 °C in Figure 6b show a similar breakdown voltage of ~1500 V (@1 × 10^−6^ A/mm) for isolation gaps of 10 µm, 20 µm, 50 µm, and 100 µm, indicating that the buffer breakdown voltage is determined by the GaN buffer layer rather than the isolation gaps. Therefore, the lateral buffer breakdown voltage is approximately two times the vertical buffer breakdown voltage, indicating that the vertical GaN buffer layers withstand a voltage of ~750 V, which is slightly lower than the value extracted from the vertical leakage measurement in Figure 6a because of a bigger ohmic contact area in the lateral measurement structure. Figure 6a,b also show the vertical and lateral leakage behavior of the epitaxial stack at 150 °C. At 150 °C, the stack’s sole vertical GaN buffer layers can still withstand a breakdown voltage of ~550 V. Meanwhile, as shown in Figure 7, we tested the voltage values at multiple positions on the GaN-on-SOI structure with RSSL buffer layers and various isolation spacings at 25°C and 150°C, when the current reached 1 × 10^−6^ A/mm. It can be observed that the voltage deviation between measurements at the same electrode spacing does not exceed 5%, demonstrating good consistency. A 750 V buffer breakdown voltage at 25 °C is used to benchmark our work against other works, as shown in Figure 8.

Figure 8 shows the breakdown voltage of GaN grown on different substrates with different buffer layer thicknesses. It is seen that the breakdown voltage of the buffer layer is almost linearly related to the buffer layer thickness, and the slope reflects the buffer layer’s resistance to voltage. It is clearly visible from the figure that the GaN-on-GaN has the strongest resistance to voltage, and the breakdown electric field of the GaN homoepitaxial buffer layer is 2.3 MV/cm, close to the theoretical value of 3 MV/cm for GaN breakdown field. However, GaN homojunction epitaxy requires a huge cost, which is not the optimal choice for the substrate. To achieve commercialization, the price is still a key factor. For GaN-on-Si with a relatively low cost, the breakdown field strength of the GaN-on-Si buffer layer is about 1.33 MV/cm, which does not maximize the advantages of GaN material. The GaN-on-SOI epitaxy layer prepared on the 200 mm SOI substrate in this work has a breakdown field strength of about 2.27 MV/cm on the epitaxy buffer layer, which is inferior to the breakdown field strength of the GaN-on-GaN epitaxy layer but better than the breakdown field strength of the GaN-on-Si epitaxy buffer layer.

**Figure 9:**
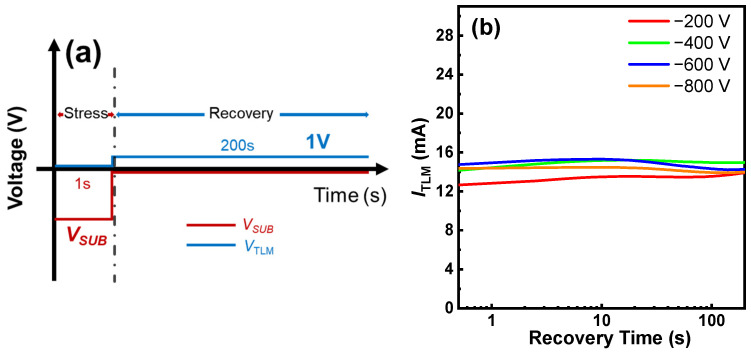
(**a**) Test procedure of buffer dispersion and (**b**) TLM current (ITLM) recovery curves after various stressing.

Buffer dispersion was then evaluated by first stressing the buffer through the substrate and then monitoring the recovery of the current of a 2DEG TLM structure [27], as shown in Figure 9a,b. The GaN-on-SOI buffer demonstrated a quite stable ITLM with a dispersion of less than 10%, meaning that the buffer is good enough for further processing for device and circuit fabrication.

**Figure 10:**
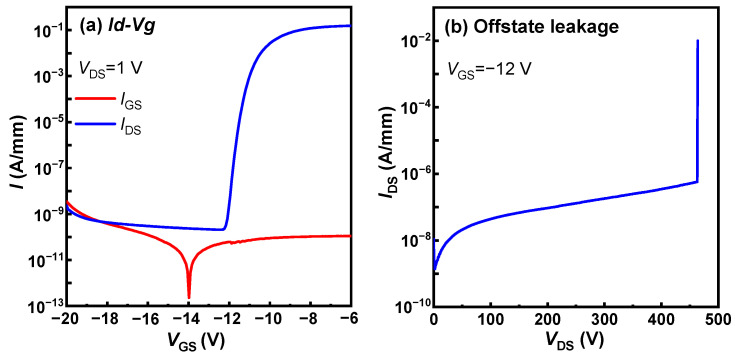
(**a**) Logarithmic transfer characteristics of a GaN power HEMT with an LGD of 6 µm on an 8-inch GaN-on-SOI substrate, measured at V_ds_ = 1V; (**b**) off-state breakdown curve, measured at V_gs_ = −12V.

Figure 10a shows the transfer characteristics of a GaN HEMT on this RSSL buffer 8-inch SOI, with a threshold voltage of approximately −11 V (@1 × 10^−4^ A/mm). Thanks to the low vertical buffer leakage of the epitaxial stack structure and the low contact resistance of the source/drain ohmic contacts, the transistor exhibits a good on/off current ratio of about 10^9^. Figure 10b shows that under off-state conditions (@Vgs = −12 V), the device leakage remains at a low level (<1 × 10^−6^ A/mm) until the breakdown voltage at around 450 V.

## 4. Conclusions

In this work, a GaN-on-SOI buffer of the reverse-stepped superlattice structure was prepared on 200 mm SOI substrates. It is seen that by increasing the Al percentage from SL1 to SL2, the slope of the growth curvature was changed from negative in SL1 to positive in SL2, indicating that the RSSL buffer introduces tensile stress instead of accumulative compress stress in a conventional stepped superlattice stack. This reverse-stepped superlattice buffer resulted in a well-controllable in situ wafer curvature, and the warpage of the wafer was less than ±50 µm for a 3.3 µm thick buffer. A 750 V breakdown voltage was achieved on the GaN-on-SOI stack, and the breakdown field strength of the epitaxial GaN buffer layer is estimated to be about 2.27 MV/cm, which is better than the breakdown field strength of the GaN-on-Si epitaxial buffer layer. The buffer in this work also demonstrated a buffer dispersion of less than 10%, indicating that the buffer is promising for further processing in device and circuit manufacturing. The fabricated device on this RSSL buffer has a good on/off ratio of ~10^9^ and can withstand a breakdown voltage of 450 V.

## Figures and Tables

**Table 1:** Warp in X, Y, and central directions.

Directions	Bowing Value [µm]
Bowing X	11.947
Bowing Y	−44.812
Center Bowing	17.135

**Table 2:** XRD test of GAN-ON-SOI samples.

Positions	GaN (002)	GaN (102)
Left-up	590 arcsec	756 arcsec
Left-down	591 arcsec	792 arcsec
Right-up	595 arcsec	919 arcsec
Right-down	594 arcsec	766 arcsec

## Data Availability

The original contributions presented in the study are included in the article, further inquiries can be directed to the corresponding author.
